# Supplementary material for: MetaRibo-Seq measures translation in microbiomes
Source: Nat Commun. 2020 Jun 29;11:3268. doi: 10.1038/s41467-020-17081-z (PMC7324362; doi:10.1038/s41467-020-17081-z)
Supplement: Supplementary file 10 — Supplementary Data 7 [file 41467_2020_17081_MOESM10_ESM.zip › File2/Confidence_High_Taxonomy/178987_out.krona.html]

Javascript must be enabled to view this page.

members
magnitude
magnitudeUnassigned
count
unassigned
taxon
rank

178987\_out

61

superkingdom
61
2

61
phylum
976

61
class
200643

171549
order
61

171550
family
61

61
genus

SRS015217\_contig\_number\_contig-100\_946.127144SRS1041138\_contig\_number\_13738SRS1055043\_contig\_number\_contig-100\_1676.128874SRS892882\_contig\_number\_15229
239759
4

species
2
1118061

SRS075821\_contig\_number\_2827SRS1041132\_contig\_number\_7492

1872444

SRS012849\_contig\_number\_20628SRS013940\_contig\_number\_21114SRS014415\_contig\_number\_1786SRS014979\_contig\_number\_36119SRS015590\_contig\_number\_10296SRS016203\_contig\_number\_9136SRS016335\_contig\_number\_49163SRS017821\_contig\_number\_32183SRS018817\_contig\_number\_23071SRS019285\_contig\_number\_109SRS043768\_contig\_number\_24484SRS043841\_contig\_number\_contig-100\_2.71468SRS044535\_contig\_number\_18686SRS046068\_contig\_number\_4583SRS047014\_contig\_number\_3807SRS047433\_contig\_number\_14024SRS048870\_contig\_number\_13884SRS049712\_contig\_number\_22940SRS050520\_contig\_number\_1264SRS054352\_contig\_number\_20315SRS056259\_contig\_number\_29639SRS063190\_contig\_number\_14172SRS065504\_contig\_number\_39698SRS075878\_contig\_number\_19914SRS076756\_contig\_number\_10360SRS076804\_contig\_number\_20635SRS077231\_contig\_number\_25736SRS077392\_contig\_number\_26673SRS098571\_contig\_number\_28815SRS098881\_contig\_number\_24879SRS1041130\_contig\_number\_6256SRS1055099\_contig\_number\_11696SRS143181\_contig\_number\_14660SRS143876\_contig\_number\_2370SRS148319\_contig\_number\_2605SRS150029\_contig\_number\_19869SRS893341\_contig\_number\_15522SRS893366\_contig\_number\_22627SRS893373\_contig\_number\_24551
39
species

2
species
28117

SRS015960\_contig\_number\_12208SRS065397\_contig\_number\_14295

species
14

SRS019693\_contig\_number\_1943SRS019968\_contig\_number\_17943SRS024388\_contig\_number\_12119SRS046369\_contig\_number\_15816SRS051882\_contig\_number\_20994SRS055017\_contig\_number\_37540SRS075341\_contig\_number\_20476SRS1041091\_contig\_number\_20365SRS104311\_contig\_number\_7543SRS104400\_contig\_number\_856SRS105082\_contig\_number\_22013SRS143417\_contig\_number\_26178SRS144183\_contig\_number\_41671SRS147271\_contig\_number\_37689
1263036
